# Cultural adaptation: a framework for addressing an often-overlooked dimension of digital health accessibility

**DOI:** 10.1038/s41746-021-00516-2

**Published:** 2021-10-01

**Authors:** Jayson S. Marwaha, Joseph C. Kvedar

**Affiliations:** 1 Beth Israel Deaconess Medical Center, Boston, MA USA; 2 Harvard Medical School, Boston, MA USA; 3 Mass General Brigham, Boston, MA USA

**Keywords:** Outcomes research, Psychiatric disorders

## Abstract

Relatively little is known about how to make digital health tools accessible to different populations from a cultural standpoint. Alignment with cultural values and communication styles may affect these tools’ ability to diagnose and treat various conditions. In this Editorial, we highlight the findings of recent work to make digital tools for mental health more culturally accessible, and propose ways to advance this area of study.

In recent years there has been tremendous growth in the availability of patient-facing digital health tools. Mental health has seen considerable activity: the US FDA has cleared a number of mental health-related digital therapeutics already this year^1,2^. The accessibility of these tools has been extensively discussed in the context of technology and infrastructure; many have raised concerns regarding access to smartphones, broadband internet, and technology literacy^3^. Cultural differences, however, have not received as much attention as an accessibility consideration.

In their systematic review, Spanhel et al.^4^ call attention to cultural adaptation as an important dimension of digital health accessibility for mental health-related tools. They distill various attempts to culturally adapt digital health tools to new target populations down to a comprehensive list of considerations. They included 55 studies that performed cultural adaptation of mental health-related digital health tools, and from these studies identified 17 essential components of cultural adaptation (Table 1). On average, these studies met 11.6 of the 17 adaptation criteria. Surprisingly, they found that there was no association between the extent of adaptation performed and the effectiveness or adherence of the digital health tool.

**Table 1:** Cultural adaptation considerations.

Content	Methods	Procedures
1. Illustrated characters	1. Structure and length of intervention	1. Method of obtaining information
2. Illustrated activities	2. Functionality and simplicity of intervention	2. Persons involved in tool development
3. Illustrated environment/burdens	3. Design and aesthetics of tool	3. Theoretical framework of tool and intervention
4. Illustrated values/traditions	4. Amount and style of guidance provided by tool	–
5. Language translation	–	–
6. Language simplification	–	–
7. Visualization of language	–	–
8. Difference in concepts of mental health	–	–
9. Goals of treatment	–	–
10. Methods of treatment	–	–

17 components of culturally adapting a digital health tool to a new target population, as identified by Spanhel et al., categorized into three dimensions: content considerations, methodological considerations, and procedural considerations.

## The scope and significance of the Issue

The authors defined 17 important criteria for adapting mental health-related tools to new target populations. However, they found that studies performing cultural adaptation efforts on their digital health tools typically only considered 68% of these criteria, and only 42% of studies included complete details of their adaptation process. While these findings alone suggest that cultural adaptation is an often-overlooked dimension of accessibility, the scope of this problem is likely even larger: there are undoubtedly many other studies – as well as unpublished digital health-based interventions – that did not meet this review’s inclusion criteria because they made no acknowledgement of, or attempt at, cultural adaptation. The performance metrics of existing tools that do not perform any cultural adaptation – or are not validated on different target populations – may be distorted.

The clinical significance of cultural adaptation is also likely understated. We were surprised that the authors found no association between adaptation and patient outcomes because there is evidence elsewhere in the literature that, when focusing on in-person mental health interventions, effectiveness is proportional to the degree of adaptation^5–8^. We would expect to see the same trends when examining digital interventions, but this work needs to be done.

Finally, as the authors state, cultural adaptation is important when applying digital health tools developed in high-income countries to populations in low- and middle-income countries. It is likely also relevant, however, when targeting different populations within diverse countries like the US, and when adapting tools designed for conditions beyond mental health as well.

## The path forward

This study helps to identify several unanswered questions that must be addressed to better characterize the role that cultural adaptation should play in digital heath.

1. To what degree is cultural adaptation capable of improving patient outcomes? This review’s findings should not diminish the importance of cultural adaptation, but rather prompt further robust head-to-head studies in more controlled settings.
2. What additional considerations exist for AI-based interventions trained on a culturally-distinct population? In addition to translating the language, aligning cultural priorities, etc. – how must we further adapt a tool that makes treatment decisions informed by a non-representative population?
3. With what granularity must adaptation be performed – for different ethnic groups, or for more specific distinctions?

Once these questions have been answered, a standardized framework for cultural adaptation will be needed to help tools achieve their optimal effect size; Spanhel et al. make progress towards this goal. The authors also lay the groundwork for repeating their systematic review so cultural adaptation best practices can be continually improved upon as the field advances^9^; new attempts to culturally adapt should be regularly reevaluated using their proposed approach.

Cultural adaptation in digital health is often poorly-reported and not done in a standardized fashion. This review represents an important, early attempt to improve the quality of an often-overlooked dimension of digital health accessibility. While more robust evidence is needed to better understand how and when cultural adaptation should be performed, digital health developers should use these 17 considerations as a guide when deploying their tools in new populations.
